# A Single Mutation in the Outer Lipid-Facing Helix of a Pentameric Ligand-Gated Ion Channel Affects Channel Function Through a Radially-Propagating Mechanism

**DOI:** 10.3389/fmolb.2021.644720

**Published:** 2021-04-30

**Authors:** Alessandro Crnjar, Susanne M. Mesoy, Sarah C. R. Lummis, Carla Molteni

**Affiliations:** ^1^Physics Department, King's College London, London, United Kingdom; ^2^Department of Biochemistry, University of Cambridge, Cambridge, United Kingdom

**Keywords:** pentameric ligand-gated ion channels, 5-HT_3_ receptors, Cys-loop receptors, mutagenesis, molecular dynamics, M4 helix

## Abstract

Pentameric ligand-gated ion channels (pLGICs) mediate fast synaptic transmission and are crucial drug targets. Their gating mechanism is triggered by ligand binding in the extracellular domain that culminates in the opening of a hydrophobic gate in the transmembrane domain. This domain is made of four α-helices (M1 to M4). Recently the outer lipid-facing helix (M4) has been shown to be key to receptor function, however its role in channel opening is still poorly understood. It could act through its neighboring helices (M1/M3), or via the M4 tip interacting with the pivotal Cys-loop in the extracellular domain. Mutation of a single M4 tyrosine (Y441) to alanine renders one pLGIC—the 5-HT_3A_ receptor—unable to function despite robust ligand binding. Using Y441A as a proxy for M4 function, we here predict likely paths of Y441 action using molecular dynamics, and test these predictions with functional assays of mutant receptors in HEK cells and *Xenopus* oocytes using fluorescent membrane potential sensitive dye and two-electrode voltage clamp respectively. We show that Y441 does not act via the M4 tip or Cys-loop, but instead connects radially through M1 to a residue near the ion channel hydrophobic gate on the pore-lining helix M2. This demonstrates the active role of the M4 helix in channel opening.

## 1. Introduction

Pentameric ligand-gated ion channels (pLGICs) are neuroreceptors involved in fast synaptic transmission underlying the physiological processes of muscle action, gut activity, and neurological function. They are present throughout the central and peripheral nervous systems, and mediate the action of biologically active compounds including nicotine, alcohol, and many anesthetics (Nemecz et al., 2016). Their wide range of functions makes them an attractive therapeutic target, if we can understand and modulate their structure and function.

While the transmission of the mechanical signal triggered by agonist binding that culminates in channel opening is not yet fully understood, significant advances can be achieved by means of mutagenesis experiments to pinpoint key residues/mechanisms as well as molecular simulations (Crnjar et al., 2019b), especially because an increasing number of high-resolution structures are now available in a variety of states (Hilf and Dutzler, 2008; Bocquet et al., 2009; Althoff et al., 2014; Hassaine et al., 2014; Miller and Aricescu, 2014; Sauguet et al., 2014; Du et al., 2015; Huang et al., 2015; Kudryashev et al., 2016; Nys et al., 2016; Basak et al., 2018a,b; Polovinkin et al., 2018; Zhu et al., 2018).

**Figure 1:**
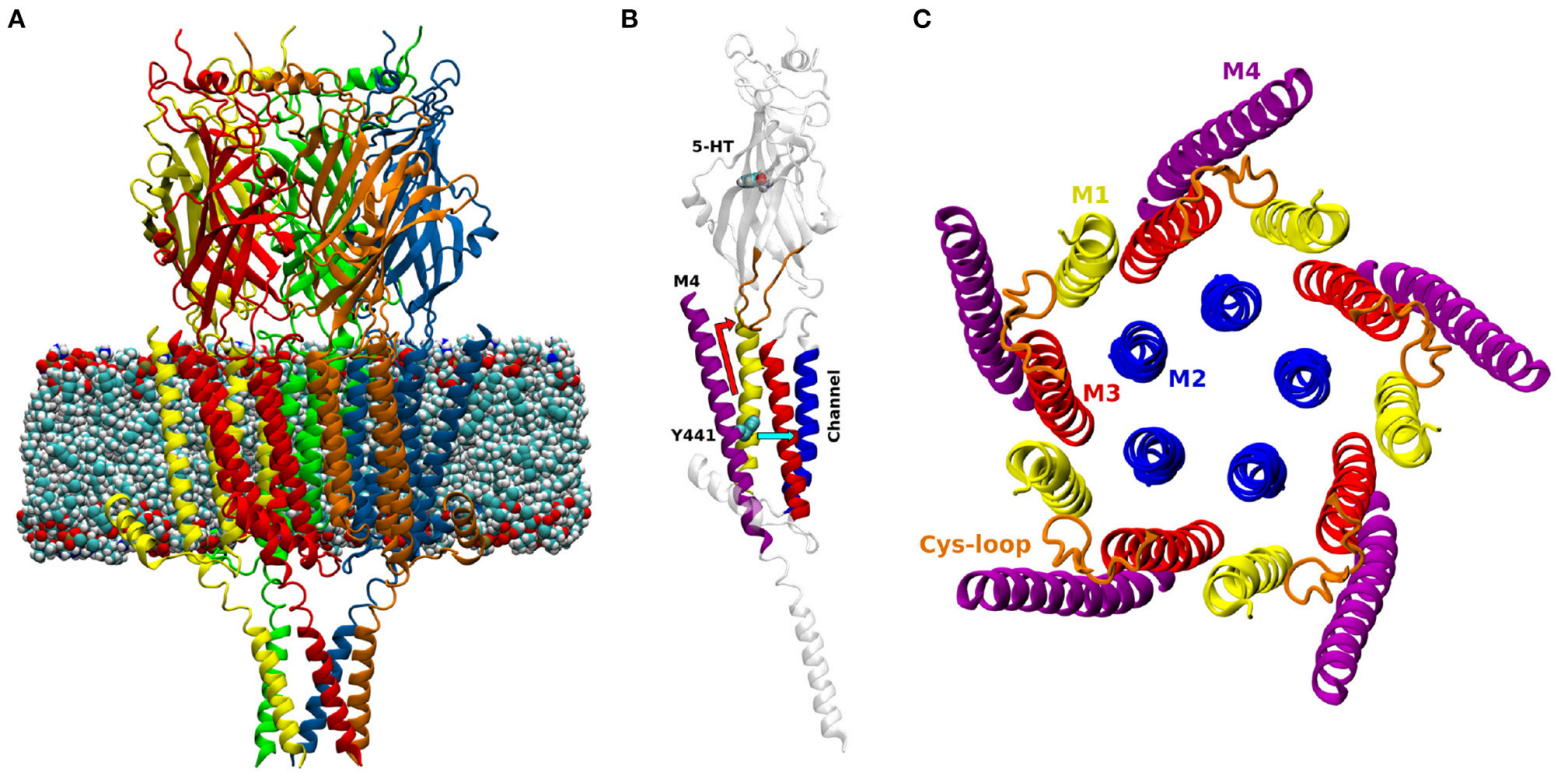
**(A)** Model of the open structure of the 5-HT_3A_ receptor (PDB ID 6DG8, Basak et al., 2018a) embedded in a lipid membrane. The five subunits are colored in red, yellow, green, blue and orange. **(B)** One subunit of the 5-HT_3A_ receptor; M1: yellow, M2: blue, M3: red, M4: purple, Cys-loop: orange. The atoms of 5-HT and residue Y441 are shown as spheres. The two possible paths of Y441A action are shown in light blue (*radial*) and red (*vertical*). **(C)** View orthogonal to the channel axis of the transmembrane domain of the 5-HT_3A_ receptor.

PLGICs are made up of five subunits, with a predominantly β-sheet extracellular domain (ECD), an α-helical transmembrane domain (TMD, containing the ion-permeable pore), and often an intracellular domain (ICD) (Figure 1). Neurotransmitters bind at the interface between subunits in the ECD, causing the channel (over 60 Å away) to open, allowing ions into the cell (Lemoine et al., 2012; Nemecz et al., 2016). The TMD is made up of four α-helices, (M1 to M4), with M2 lining the channel pore, M1 and M3 in a second concentric circle, and finally M4 facing the lipid membrane (Figure 1C). While this helix is fundamentally an amphipathic barrier to the hydrophobic lipid environment, there is growing evidence that the M4 helix plays a key role in pLGIC function: mutations in the M4 helices of mammalian pLGICs have been shown to reduce or inhibit channel opening (Cory-Wright et al., 2018; Tang et al., 2018; Mesoy et al., 2019), or promote channel function (da Costa Couto et al., 2020) although the exact mechanism is not yet clear.

There are two main proposed mechanisms for M4 affecting channel opening. One mechanism, first outlined by Da Costa et al. (DaCosta and Baenziger, 2009) proposes that the C-terminal end of the M4 helix, which sits at the level of the ECD-TMD interface, is required for the signal transduction through that interface via interactions with the Cys-loop. Loss of the M4 tip or of M4 binding to the rest of the TMD would disrupt this interface. This would not affect ligand binding, but would prevent the channel opening signal from reaching the TMD, resulting in what has been described as an uncoupled receptor. In this case, mutations that alter the pinning of M4 to the rest of the channel could disrupt M4/Cys-loop interactions in what we term a “vertically”-propagating chain of events (i.e., propagating along a direction parallel to the protein axis). This model is structurally appealing, and supported by the fact that allosteric modulation has been shown to propagate from M4 tip residues to the Cys-loop in the cationic α4β2 nicotinic acetylcholine receptor (nAChR) (Alcaino et al., 2017).

The other mechanism would involve residues of the M4 acting directly on M1/M3, i.e., “radially,” and this signal propagating to affect channel opening, e.g., by interactions with M2. A naturally occurring M4 mutation (C418W) in the *Torpedo* nAChR which alters channel function has been shown to be energetically coupled to two M1 residues (S226 and T229) (Domville and Baenziger, 2018). The same work also shows that the C418W mutations does not affect interactions of the M4 C-terminal domain (CTD) with the Cys-loop, supporting the radial mechanism proposal.

In addition to functional characterization showing the involvement of M4 in channel activity, the M4 of the neuromuscular nAChR has been calculated to move as a unit approximately halfway through receptor activation, further supporting its possible role in connecting ligand binding (the first event of activation) to channel opening (Mitra et al., 2004). Together this experimental and theoretical work highlights an intriguing role for the M4 helix in coupling channel opening to ligand binding, which could be influenced by mutations and by the lipid membrane (e.g., its composition, thickness, or fluidity).

Among pLGICs, the 5-HT3A receptor (5-HT_3A_R) constitutes a unique case when it comes to the mechanism of action of the M4 helix. Alanine mutation of a single tyrosine (Y441), located approximately at the center of the M4 (Figure 1B) prevents receptor activation but not ligand binding. Of the other 26 M4 residues tested (434-461), 24 can be changed to alanine with little or no detectable effect on channel function (the remaining two abolish receptor expression, so their effect on function is unclear) (Mesoy et al., 2019). We take this to mean that Y441-dependent coupling can be taken as a proxy for the coupling mediated by the entire M4 helix in this channel. Here we use the uncoupling Y441A mutation to probe the role and function of the 5-HT3AR M4 helix by comparing it to the wild-type (WT) in molecular dynamics (MD) simulations, testing proposed mechanisms by mutagenesis and functional assays.

## 2. Materials and Methods

### 2.1. Molecular Biology

The mouse 5-HT3A cDNA (Q6J1J7) in pcDNA3.1 (for HEK cell transfection) or pGEMHE (for RNA production) was modified by QuikChange site-directed mutagenesis (Agilent Technologies) to create point mutants (verified by sequencing).

### 2.2. Cell Culture

Human embryonic kidney (HEK) 293 cells were maintained at 37^o^C at 5% CO_2_ in a humidified atmosphere, in Dulbecco's Modified Eagle's Medium/Nutrient Mix F12 (1:1) (Invitrogen, Paisley, UK) with GlutaMAX^TM^ and 10% fetal bovine serum (GE Healthcare) (DMEM/FBS) and passaged when confluent. Five micrograms of WT or mutant 5-HT3A DNA and 30 μl polyethyleneamine (Polysciences) incubated for 10 min in 1 mL DMEM was added to 60% confluent HEK293 cells for transfection, and cells grown for 2 days before assays.

### 2.3. FlexStation

As described previously (Price and Lummis, 2005) cells were incubated for 45 min with fluorescent membrane potential-sensitive dye (Membrane Potential Blue kit, Molecular Devices) diluted in Flex buffer (10 mM HEPES, 115 mM NaCl, 1 mM KCl, 1 mM CaCl_2_, 1 mM MgCl_2_, and 10 mM glucose, pH 7.4), and subsequently assayed at room temperature for 180 s, with readings every 2 s. 5-HT was added to each well after 20 s. Concentration-response curves were generated by iterative fitting in GraphPad Prism 7 (after normalization to max ΔF) with the equation $y=a+\frac{b-a}{1+10^{\left({\text{n}}_{\text{H}}({\text{logEC}}_{50^{\text{-x}}})\right)}}$ where *y* is the fluorescent response, x is log[5-HT] (log of the concentration of ligand), *a* is the minimum response, *b* is the maximum response, and n_H_ is the Hill slope.

### 2.4. Radioligand Binding

This was performed as previously described. Lummis and Thompson (2013) Briefly receptors in crude HEK293 cell membranes were labeled with the 5-HT_3_R antagonist [^3^H]GR65630 by incubation in 0.5 mL 10 mM HEPES buffer pH 7.5 for 1 h on ice, using 1 μM quipazine to determine non-specific binding. Data were analyzed in GraphPad Prism by iterative curve fitting.

### 2.5. Two-Electrode Voltage Clamp

*Xenopus laevis* oocytes from EcoCyte Biosciences (Austin Texas) were injected with 100 pg cRNA (generated with the ThermoFisher mMESSAGE mMACHINE T7 transcription kit) and left in injection media [88 mM NaCl, 2.4 mM NaHCO_3_, 1 mM KCl, 0.82 mM MgSO_4_ ·7H_2_0, 5 mM Tris-HCl, 0.33 mM Ca(NO_3_)2·4H_2_O, 0.41 mM CaCl_2_·2H_2_O, 2.51 mM sodium pyruvate, 0.12 mg/ml theophylline, 0.05 mg/ml gentamicin, pH 7.5] at 16^o^C for 24 h. Recording was performed at 22^o^C on a Roboocyte (Multichannel systems, Reulingeen, Germany), using calcium-free ND96 buffer (96 mM NaCl, 2 mM KCl, 1 mM MgCl_2_, 5 mM HEPES, pH 7.5) and 5-HT solutions applied by a computer-controlled perfusion system. The holding potential was −60 mV, using glass microelectrodes with a resistance of approximately 1 MΩ backfilled with 3M KCl.

### 2.6. Model and Molecular Dynamics Simulations

A model of the 5-HT3AR was built based on the cryo-EM open structure resolved by Basak et al. at 3.89 Å resolution (pdb entry: 6DG8, Basak et al., 2018a) (Figure 1A). The open structure was chosen in part because we assumed that the M4 helices would play their role late in the overall chain of receptor activation (i.e., subsequent to ligand binding), and therefore the effect of mutating Y441A would be better observed in a receptor conformation that is at the end and not the start of the activation process. Additionally, there was experimental evidence that WT and Y441A-containing receptors showed differences with regard to the open state (the WT can attain this state but the mutant receptor does not; Mesoy et al., 2019), suggesting that the open state was more likely to reveal functional differences between the two receptors.

This structure comprises the TMD, the ECD, and part of the ICD: the highly flexible residues 333–396 in the ICD were not resolved experimentally. This unstructured region was not reconstructed, considering not only its considerable length (which would result in a large solvation box), but also its likely lack of influence on the M4 helices. Conversely, the experimentally resolved and structured part of the ICD (the MA helices) was included, as the M4 movements may in principle be affected by the MAs.

The model was protonated at neutral pH, and embedded in a 6:7:7 cholesterol-POPC-POPE lipid membrane (with lipids randomly distributed) using the CHARMM-GUI web-based membrane builder (Jo et al., 2008), resulting in a membrane area of about 124 by 127 Å. The 6:7:7 concentration was chosen to resemble HEK cells membrane composition, resulting in a cholesterol/phospholipid ratio of 0.42, closer to the value of 0.48 in HEK cells (Dawaliby et al., 2016) than to that of 0.6–0.7 in oocytes (Opekarová and Tanner, 2003). This ratio has been used for simulations of membranes with cholesterol (6), POPC (7) and a third lipid (7) for the study of serotonin receptors (Shan et al., 2012; Crnjar and Molteni, 2020; Guros et al., 2020). Mixed membranes containing POPC and POPE (together with cholesterol), have also been studied in the past (Elmore and Dougherty, 2003; Mahmood et al., 2013; Cao et al., 2015; Patra et al., 2015; Heusser et al., 2018; Oakes and Domene, 2019; Guros et al., 2020). The presence of cholesterol is important as this lipid is present in high concentration in brain cells membranes (Pfrieger, 2003; Chan et al., 2012), and a mixed membrane may prove important for a cooperative modulation of the effects of the Y441A mutation.

The system was then solvated in an orthorhombic supercell, with 52,477 TIP3P water molecules and 0.15 M of Na^+^ and Cl^−^ ions to reproduce physiological conditions, together with 5 Cl^−^ counterions to counterbalance the positive charge of the five bound 5-HT molecules. The total number of ions was 162 for Na^+^, and 142 for Cl^−^. PyMOL (Schrödinger, LLC, 2015) was used to turn the five Y441 into alanines, resulting in a mutated receptor (MR) model. In total the WTR model contained 226,082 atoms and the MR model contained 226,027 atoms.

The systems were simulated with the NAMD 2.13 molecular dynamics package (Phillips et al., 2005), the AMBER ff14SB (Maier et al., 2015) and LIPID14 force-field (Dickson et al., 2014). The five 5-HTs in the binding pockets were parameterized as described in the Supplementary Material. The simulation time step was 2 fs, and the bonds containing hydrogen were constrained with the SHAKE algorithm. Particle Mesh Ewald was employed for the electrostatic interactions and a cut off of 10 Å was used for the non-bonded interactions.

At the beginning, the WTR and MR models underwent a minimization procedure, a slow heating and a partially restrained equilibration (with the protein α carbons and the 5-HT rings restrained while the lipids were free to diffuse). The equilibration of lipid membranes requires long time windows since their diffusion occurs over times of the order of tens to hundreds of nanoseconds (Kandt et al., 2007; Smith et al., 2018). Thus, the equilibration stage, performed within the isothermal-isobaric (NPT) ensemble, lasted around 150 ns in total, while slowly releasing the chosen restraints. Supplementary Table 1 reports the full equilibration procedure followed.

After the equilibration, production runs were performed for both models, with 1.0 kcal/mol^2^ restraints on M2, MA, MX α carbons. These restraints were kept during the production, since care must be taken in order to prevent the collapse of open structures of pLGICs, including the possible closure of the channel (Dämgen and Biggin, 2020). Past simulations on this very receptor (Crnjar and Molteni, 2020; Guros et al., 2020) without any restraints applied highlighted how the RMSD of the M4 can go up to 4 Å. This is similar to what was found in our simulations, thus proving that the chosen restraints do not affect the section from residue 441 and above. Moreover, residue 425 is far outside the membrane within the ICD, as shown by Supplementary Figure 4.

The production was carried out within the isothermal-isobaric (NPT) ensemble for each model at a temperature of 310 K, which is above gel transition temperature for all lipid species (Silvius, 1982; Kraske and Mountcastle, 2001), and at a pressure of 1 atm. Temperature was controlled by means of a Langevin thermostat with a collision frequency of 1.0 ps^−1^, and pressure was controlled by means of a Langevin piston barostat with an oscillation period of 200 fs and a damping time constant of 100 fs.

For both WTR and MR, two replicas of 250 ns each were simulated, referred to in the following as R0 and R1. This choice was made as a consequence of the stochastic nature of the Langevin dynamics, which would result in different trajectories in different replicas, particularly affecting the diffusion of lipid molecules, to whom the outermost M4 helices are exposed. Most of the analysis described in the following were performed over the conjunction of the time windows 50-to-250 ns of both R0 and R1 (R01-400). In fact, to improve statistics when performing simulations, long runs or multiple replicas would give qualitative similar results for time- and subunit-averaged quantities (Crnjar and Molteni, 2020). Moreover, the pentameric nature of this pLGIC allows for a simultaneous five-fold sampling of whatever phenomenon occurs within one subunit. For both R0 and R1, the first 50 ns of simulations were excluded from statistics collection in order to mitigate for the use of the same initial geometry and allow for independent equilibration (50 ns being the time window after which the protein RMSD flattens, as shown in Supplementary Figure 1). The analysis of quantities which needed to be expressed as functions of time, or that would be dependent on the order of the union of R0 and R1, were performed separately for R0 and for R1.

Trajectories were sampled every 50 ps, and analyzed with the Cpptraj (Roe and Cheatham, 2013) and MDAnalysis (Michaud-Agrawal et al., 2011; Gowers et al., 2016) software. Hydrogen bonds were defined by using a donor-acceptor distance smaller than 3.5 Å and a donor-hydrogen-acceptor angle larger than 120°. These values have been used in several previous works on pLGICs (Melis et al., 2008; McCormack et al., 2010; Comitani et al., 2014, 2015, 2016; Crnjar et al., 2019a,b), and are the defaults of analysis software such as MDAnalysis (Michaud-Agrawal et al., 2011; Gowers et al., 2016). A generic contact between any two given atoms (of two different residues) was considered here when their distance was shorter than a cutoff of 3.5 Å as in previous studies (Deol et al., 2004).

Aromatic interactions were calculated by considering distances and angles involving the vector normal to best-fit-plane to a given single aromatic ring (for residues with multiple aromatic rings, such as tryptophan, we consider the rings separately and then sum the interactions frequencies). π-π interactions consider a ring-ring distance less than 6.0 Å, and normals angle smaller than 45° or greater than 135°. The distance was chosen in order to be 1 Å larger than the optimal one predicted for benzene dimers (Sinnokrot et al., 2002). Anion-π interactions considered a ring-charged atom distance smaller than 5.0 Å, and an angle between ring normal and ring center-to-negative atom distance smaller than 40° or greater than 140° (Lucas et al., 2016).

## 3. Results

### 3.1. Vertical Mechanism

**Figure 2:**
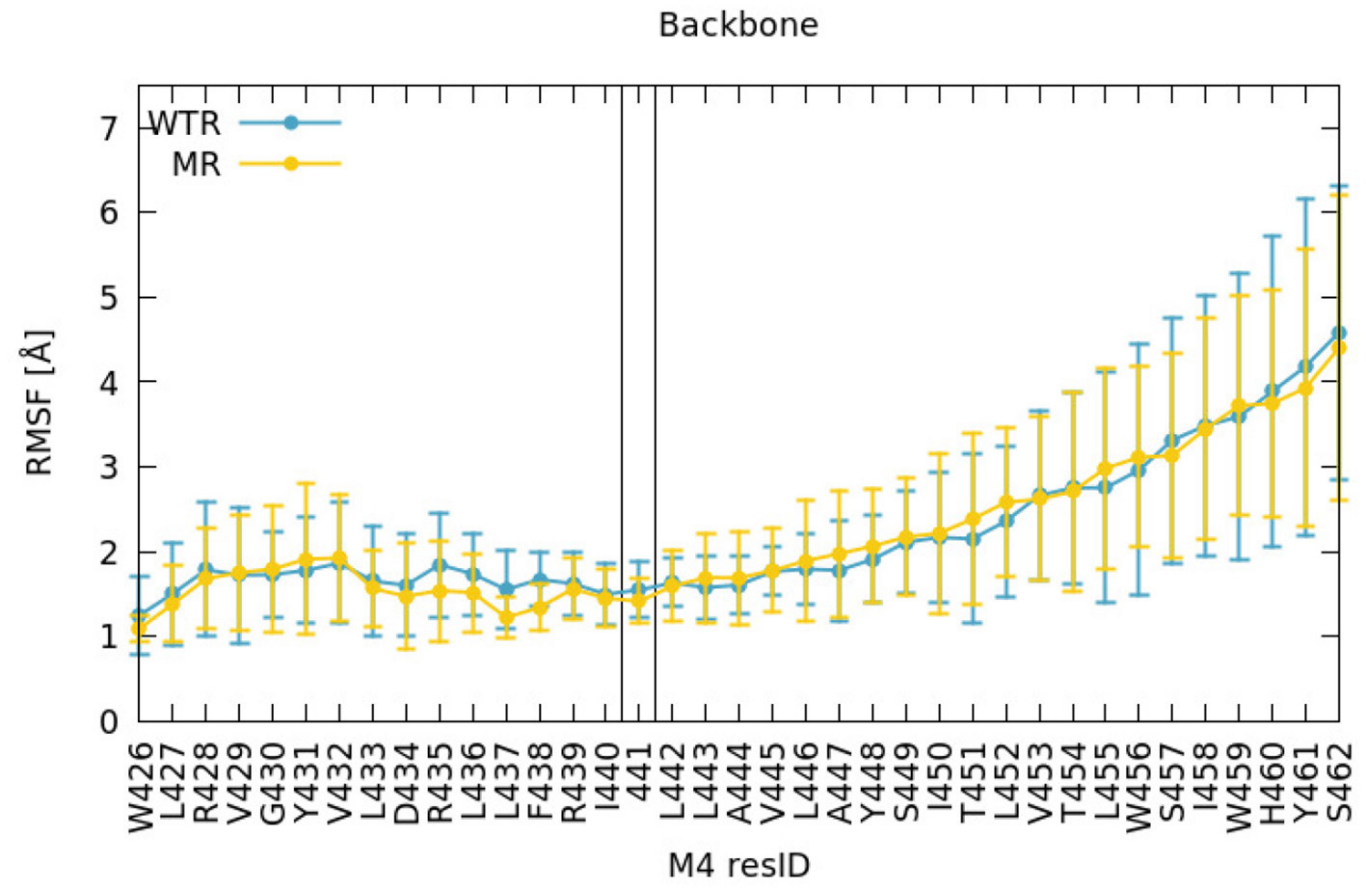
RMSF of backbone atoms of the M4 amino acids, calculated for R01-400 for the WTR (blue) and the MR (yellow).

We found no difference between the WT and mutant simulation either at or above residue 441 on M4 from examining the local dynamical fluctuations of M4 by evaluating the root mean square fluctuations (RMSF) of each residue (Figure 2). This was calculated over R01-400 with respect to the post-equilibration positions, for the backbone atoms of those residues, and averaged over each of the five subunits. The errors were calculated as maximum semidifferences (half of the difference between maximum and minimum value).

Residue 425 (the last residue of the MA helix) was restrained in both simulations, so we did not consider nearby similarities valid. Around residue 441, the fluctuations were around 1–2 Å in both WTR and MR; on average the dynamics of residue 441 did not seem to be affected by removing the side chain. The fluctuations increased (as did the variation across subunits) toward the top of the helix. The M4 tip is located at the interface with the solvent in the extracellular region, at the same level of the edge of the outer lipid leaflet (Supplementary Figure 4), and is therefore allowed to move more freely. These comments hold true for both the WTR and the MR, which show little difference in the dynamics.

**Figure 3:**
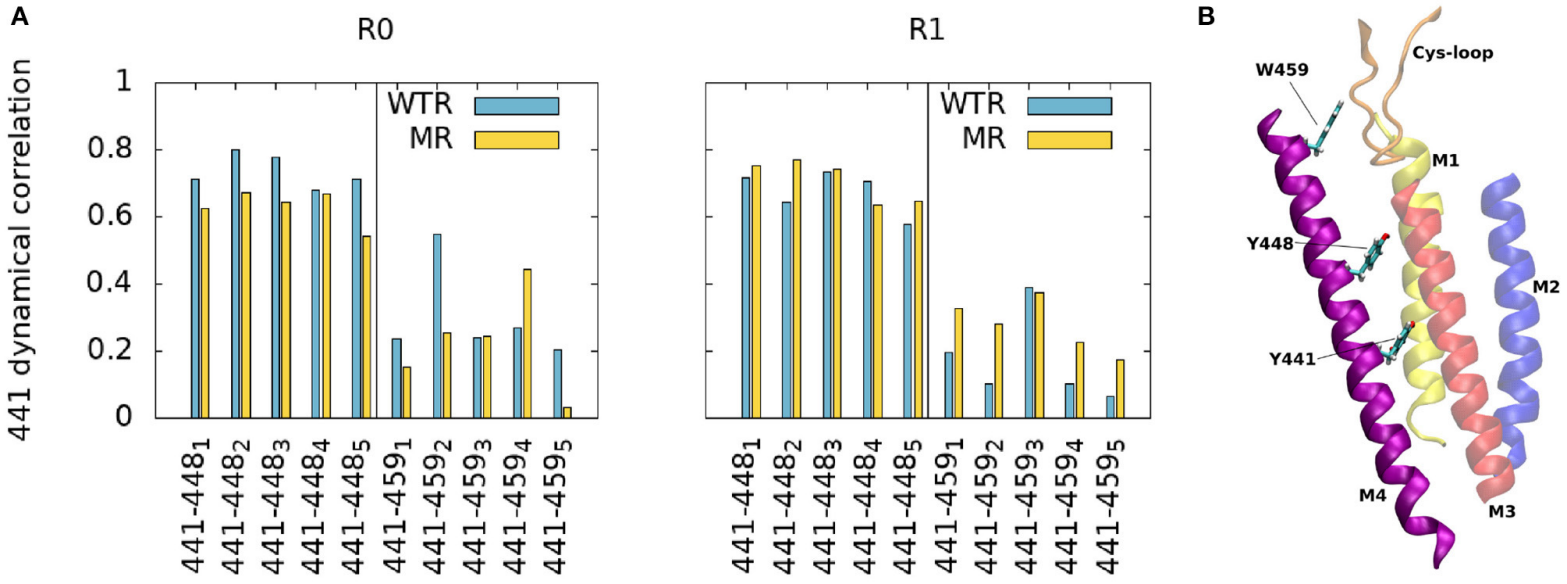
**(A)** Average dynamic correlation of residue 441 backbone atoms with those of residues 448 and 459, for the WTR (blue) and the MR (yellow) in the two replicas R0 and R1. Subscripts denote the five subunits. **(B)** Position of residues 441, 448, and 459 in the protein.

The top of the M4 helix therefore appears not to depend on the properties of residue 441. To confirm this, we investigated other factors. Firstly, to better estimate the effect of residue 441 dynamics upwards along the M4, we calculated the time-averaged dynamical correlation of this residue with respect to two representative amino-acids: Y448 and W459 (Figure 3). Both are structurally important; mutation of either residue abolishes cell surface expression (Mesoy et al., 2019). Y448 is an integral inwards-facing residue near Y441, and W459 is a good candidate for potential interactions with the Cys-loop, making it an interesting marker of M4 tip behavior. The dynamical correlation C_*ij*_ between two atoms *i* and *j* is defined (Hunenberger et al., 1995) as:

(1) $$\begin{array}{r} C_{ij}=\frac{\langle \vec{r_{i}}\vec{r_{j}}\rangle -\langle \vec{r_{i}}\rangle \langle \vec{r_{j}}\rangle }{\sqrt{\left(\langle {\vec{r_{i}}}^{2}\rangle -{\langle \vec{r_{i}}\rangle }^{2}\right)\left(\langle {\vec{r_{j}}}^{2}\rangle -{\langle \vec{r_{j}}\rangle }^{2}\right)}} \end{array}$$

C_*ij*_ may take any value between 0 and 1: values close to 1 indicate that the two residues move consistently in the same direction over time, acting like a rigid body, while values close to 0 imply that the dynamics of these residues never display any correlation.

The correlations between residues 441 and 448/459 appear to be subunit- and replica-specific, although the 441-448 correlations are consistently higher than the 441–459 ones (as expected due to residue 448 being closer than 459 to 441). While for the pair 441–448 they reach values up to 0.8 (with an average of 0.69 ± 0.07), for the pair 441–459 they never surpass 0.6 (with an average of 0.24 ± 0.13), implying that 441 is not notably correlated with the top of the M4.

**Figure 4:**
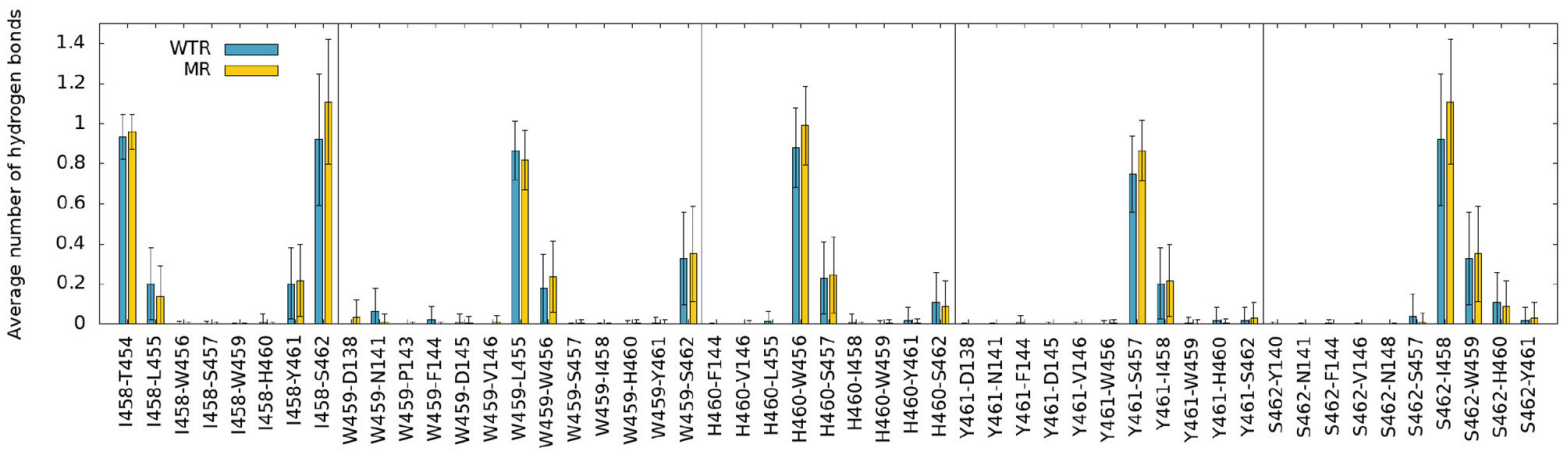
Hydrogen bonds of M4 tip residues (I458, W459, H460, Y461, S462), calculated over R01-400 for the WTR (blue) and the MR (yellow).

**Figure 5:**
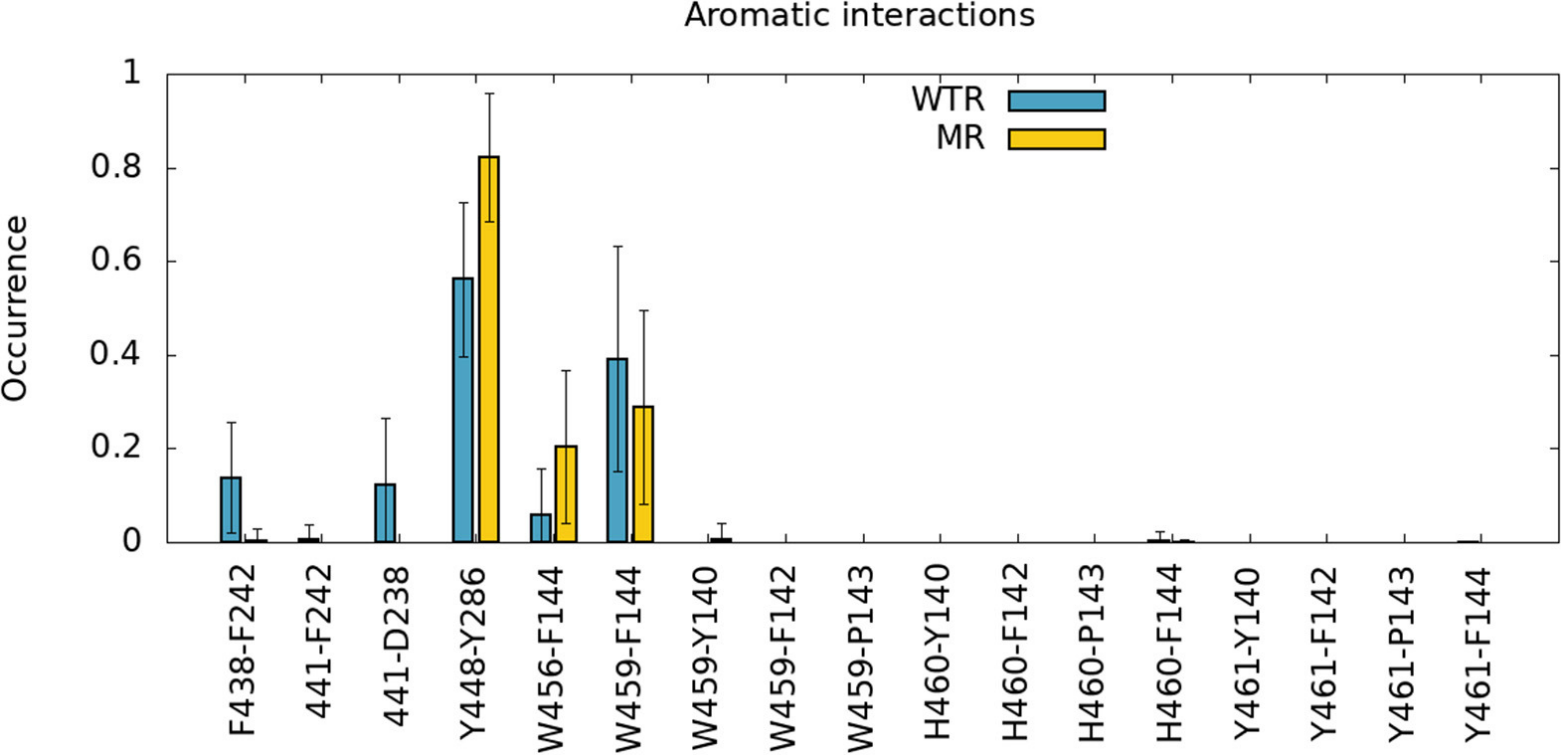
π-π interactions (F438-F424,441-F242,Y448-Y286,W456-F144,W459-F144) and anion-π interactions (441-D238) of different amino-acids of M4, calculated over R01-400 for the WTR (blue) and the MR (yellow).

Secondly, we investigated the time- and subunit-averaged interactions (hydrogen bonds, π-π interactions and anion-π interactions) of selected M4 residues, evaluated over R01-400 (Figures 4, 5). Errors were calculated via error propagation from the five standard deviations of data over time for each of the single subunits.

Figure 4 confirms that W459 is the only M4 tip residue that forms hydrogen bonds (although with low frequency) with the Cys-loop (residues 135 to 149). No major differences are noted between WTR and MR in either hydrogen bonds nor aromatic interactions, except for the fact that the removal of the 441 side chain upon mutation prevents it from forming interactions with its neighbors.

### 3.2. Radial Mechanism

**Figure 6:**
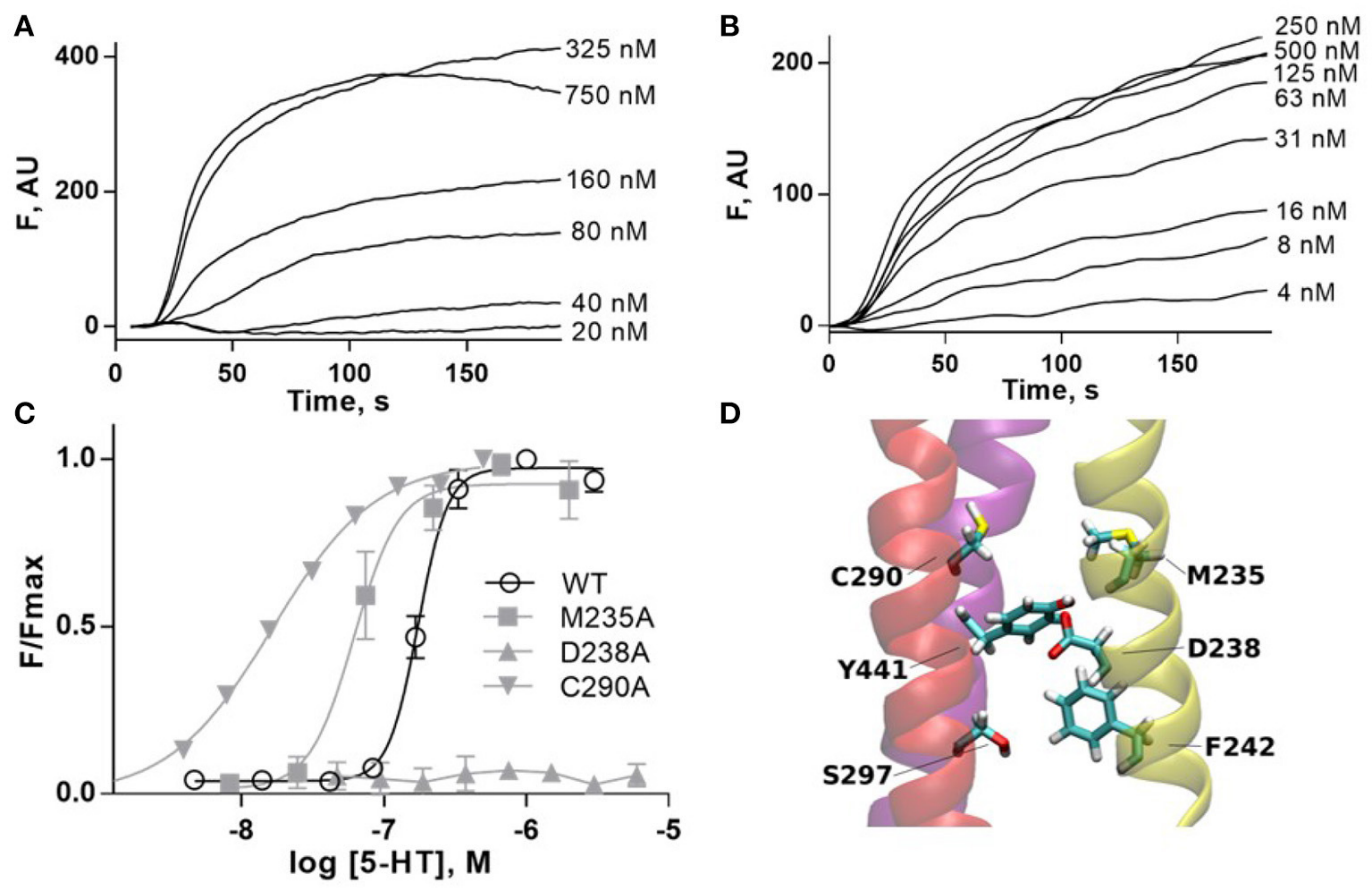
**(A,B)** Typical fluorescent responses (F in arbitrary units, AU) to addition of 5-HT at 20 s to HEK293 cells expressing 5-HT3A receptors, using a membrane potential sensitive dye. **(A)** WT, **(B)** C290A. **(C)** 5-HT concentration-response curves of mutant receptors in HEK293 cells. For D238A F is compared to WT Fmax. Data is mean ± standard error of the mean (SEM), *n* ≥ 3. **(D)** Molecular dynamics snapshot showing Y441 in the M4 helix together with possible interaction partners. M1: yellow, M3: red, M4: purple.

The radial mechanism was first investigated experimentally. We characterized WT and mutant 5-HT3A receptors expressed in HEK293 cells by measuring responses of a membrane potential-sensitive dye on addition of 5-HT (Figures 6A–C). This gave a WT EC50 of 0.17 μM (pEC50 of 6.76 ± 0.01) and a Hill slope of 3.7 ± 0.3, which is comparable to previous work (Lochner and Lummis, 2010). The WT level of ligand binding at the cell surface was measured with [^3^H]GR65630, giving a K_d_ of 0.18 ± 0.03 nM, similar to previous work e.g., (Hovius et al., 2002), and a B_max_ of 1.2 ± 0.8 pmol/mg protein (Table 1). Mutants that did not respond in the functional assay are marked as non functional (NF).

**Table 1:** Mutant receptors in HEK293 cells.

**Mutant**	**EC50 (μM)**	**pEC50 (M)**	**n_H_**	**K_d_ (nM)**	**B_max_ (pmol/mg protein)**
WT	0.17	6.76 ± 0.01	3.7 ± 0.3	0.18 ± 0.03	1.2 ± 0.8
M235A	0.06	7.21 ± 0.05	2.8 ± 0.9	0.70 ± 0.09	2.4 ± 0.4
D238A		NF		0.50 ± 0.07	1.8 ± 0.4
F242A	0.31	6.51 ± 0.02	3.8 ± 0.8		
C290A	0.02	7.79 ± 0.05	1.2 ± 0.2	0.85 ± 0.10	3.8 ± 0.3
S297A	0.46	6.34 ± 0.03	3.5 ± 0.8		

*K_d_ ± B_max_ measured by saturation binding. Values are mean and SEM, n ≥ 3*.

Alanine mutations of all residues of interest near Y441 showed that only D238A had a comparable effect to Y441A (Figure 6 and Table 1). Neither Y441A nor D238A responded to application of 5-HT, though they both had high levels of [^3^H] GR65630 binding sites (Mesoy et al., 2019 and Table 1). Their proximity and similar phenotypes on mutation indicate that D238 could be part of the mechanism of Y441 supporting channel function.

**Figure 7:**
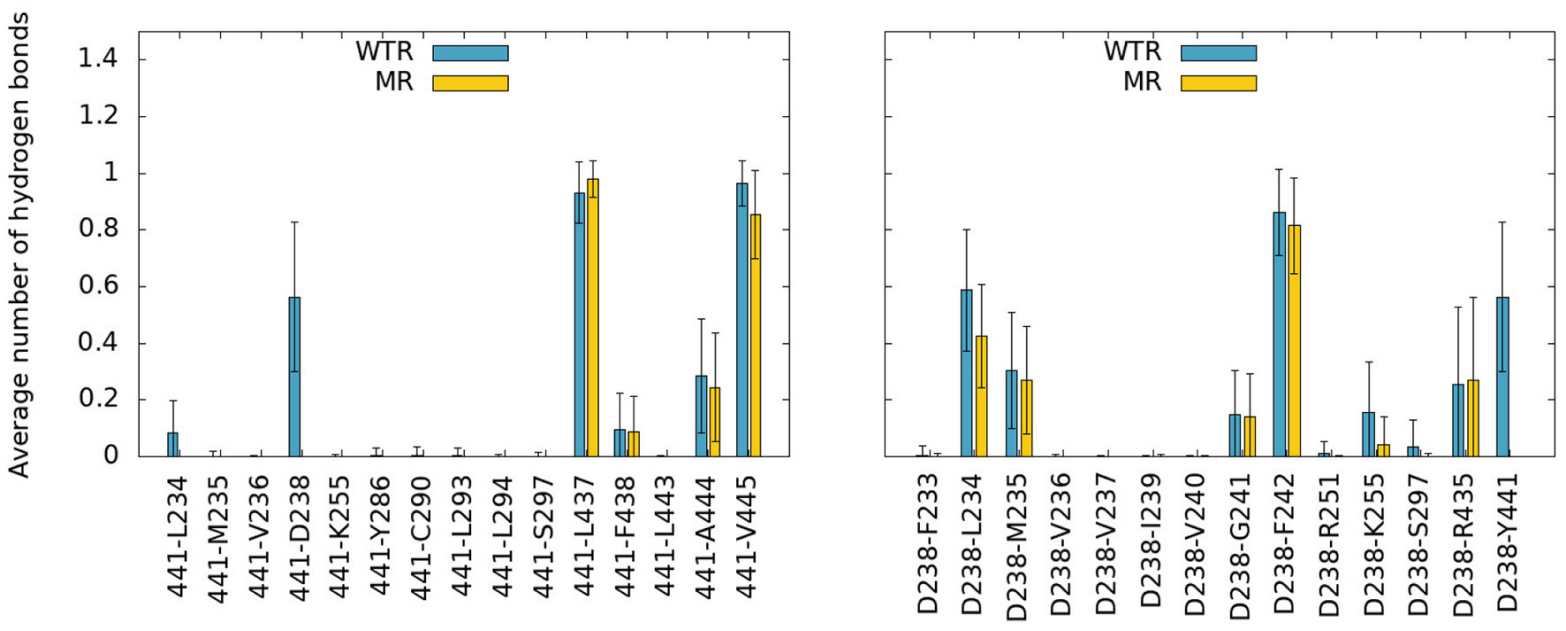
Hydrogen bonds of residue 441 **(left)** and D238 **(right)** with any other protein residue, calculated over R01-400 for the WTR (blue) and the MR (yellow).

To further elucidate the role of the Y441-D238 interaction, we investigated the wider effects of Y441A through D238. In the MD simulations, the time- and subunit-averaged hydrogen bonds of D238 and Y441 with any other residue belonging to the M1, M2, M3, and M4 helices were calculated over R01-400 (Figure 7) for both the WTR and the MR. Error bars were calculated via error propagation from the five standard deviations of time-data for each of the single subunits.

**Figure 8:**
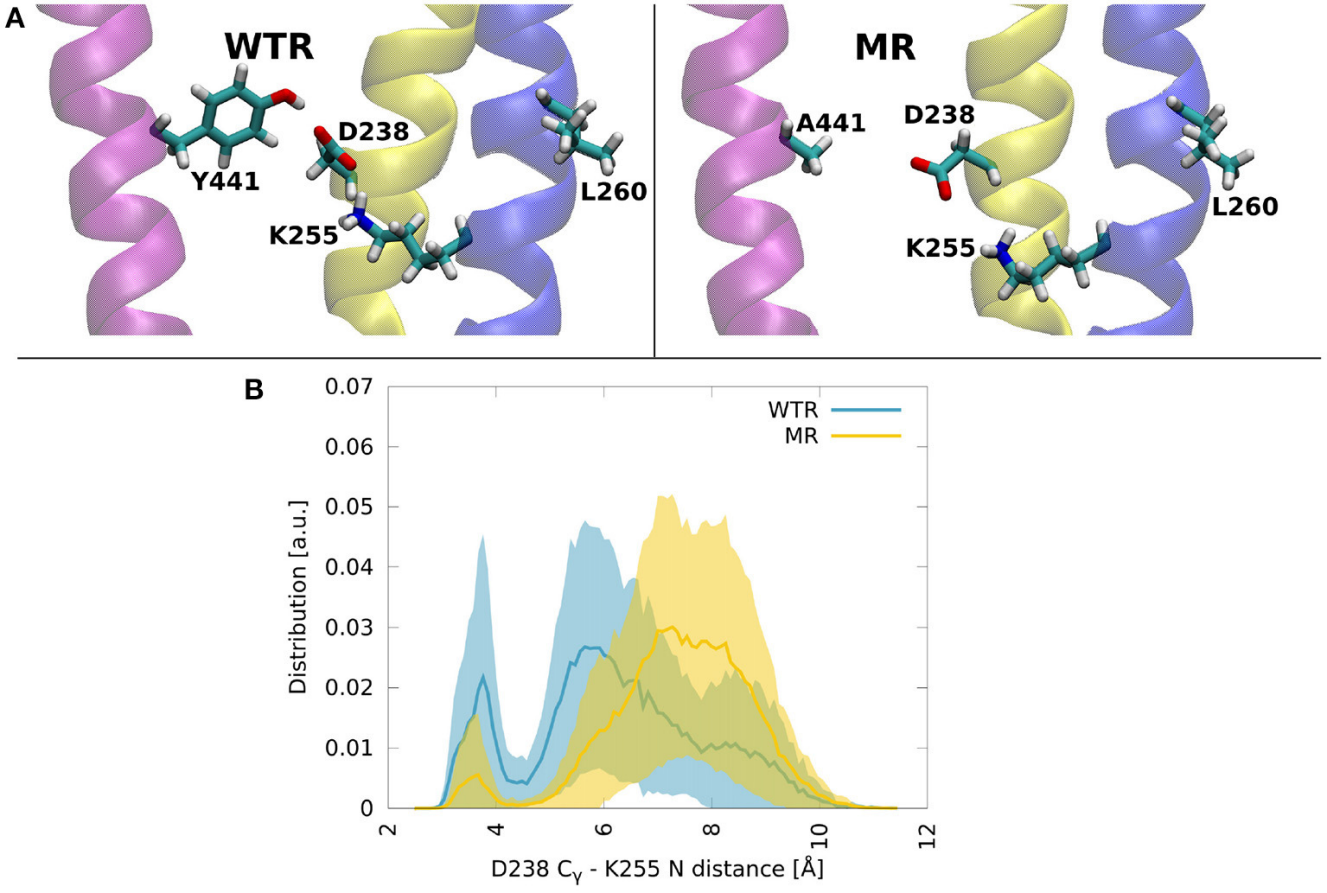
**(A)** Molecular dynamics snapshots showing how Y441 might help maintain the hydrogen bonding between D238 and K255 in the WTR, while in the MR these two residues are less likely to form interactions. M1: yellow, M2: blue, M4: purple. **(B)** Distributions of the distance between C_γ_ of D238 and the terminal nitrogen of K255, in the two models. Thick lines: averages; shaded area: error.

No major differences were observed between the two models, except for the notable lack of hydrogen bonds between residues 441 and 238 in the MR. However, one interesting fact emerges: both residues 238 and 441 are able to make interactions with K255, a lysine that belongs to the M2 helix. This residue is near L260, the hydrophobic gate of the 5-HT_3_R (Hassaine et al., 2014; Aryal et al., 2015). The K255 side chain stretches between M1 and M3 and points toward the middle of the four TMD helices, with its terminal nitrogen at a convenient position for the formation of hydrogen bonds with residues in the region (Figure 8A).

Residue 441 is within reach of the K255 side chain, so direct hydrogen bonds between these two residues are possible, however they only occurred for a tiny fraction of the simulation time. Conversely, D238 terminal oxygens formed hydrogen bonds for longer.

The distribution over time and subunits of the distance between the C_γ_ of D238 and the terminal nitrogen of K255 revealed two peaks in both the WTR and the MR (Figure 8B). Errors were calculated via error propagation from the ten standard deviations of data over time for each of the single subunits and for the two replicas R0 and R1. One peak was found at about 7.5 Å for the MR and at about 5.5 Å for the WTR, and shows how the absence of Y441 side chain allows for D238 to be farther away from K255 with respect to the WTR. Another peak was observed around 3.5 Å for both the WTR and the MR, and was much higher for the WTR than for the MR: this indicates that a hydrogen bond may be formed between K255 and D238, which was observed for longer times in the WTR. In the WTR, the distance between C_γ_ of D238 and the side chain oxygen of Y441 is 4.8 ± 0.4 Å.

To investigate this putative interaction we assayed the effects of mutating K255 in HEK293 cells. K255A is indistinguishable from WT, indicating that K255 is not required for correct channel function. Intriguingly however, K255L is entirely non-responsive to ligand, even though it is expressed, as shown by radioligand binding (Table 2). This indicates that K255 may be part of the same interaction chain as Y441 and D238.

**Table 2:** Mutant receptors in HEK293 cells.

**Mutant**	**EC50 (μM)**	**pEC50**	**n_H_**	**K_d_ (nM)**	**B_max_ (pmol/mg protein)**
WT	0.17	6.76 ± 0.01	3.6 ± 0.3	0.18 ± 0.03	1.2 ± 0.8
K255A	0.52	6.29± 0.02	2.6± 0.1		
K255L		NF		0.17± 0.02	0.4 ± 0.2
K255Q	0.11	6.95± 0.02	1.9± 0.2		
K255E	0.26	6.59± 0.02	4.4± 1.4		
K255C	0.30	6.52± 0.03	2.3± 0.6		

*K_d_ and B_max_ measured by saturation binding. Values are mean and SEM, n ≥ 3*.

### 3.3. Rescue of Non-functional Receptors

We decided to further probe the most interesting mutants in a different expression system, *Xenopus* oocytes, using two-electrode voltage clamp.

**Figure 9:**
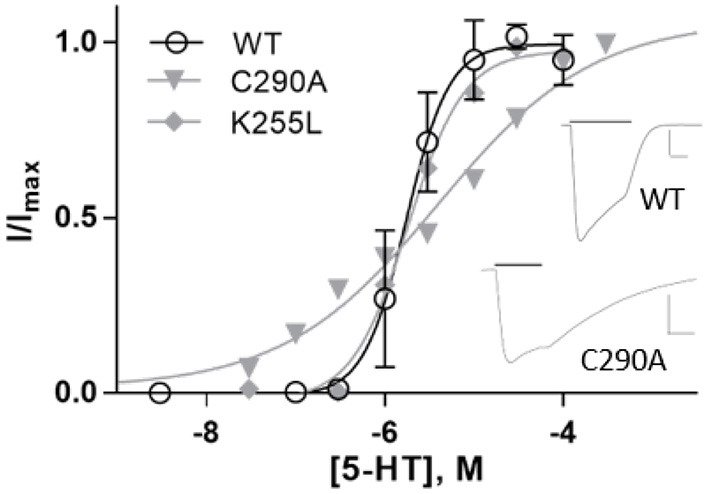
Concentration-response curves of 5-HT3A receptors in *Xenopus* oocytes. Data is mean ± SEM, n≥3. Inset: typical current recordings at 3 μM 5-HT; scale bars are 20 s and 2 μA.

Expressing WT 5-HT3A in *Xenopus* oocytes gave an EC50 of 1.7 μM (pEC50 = 5.76 ± 0.05) and a Hill slope of 1.8 ± 0.3 (Figure 9 and Table 3), similar to previous work (Lummis et al., 2016).

**Table 3:** Mutant receptors in *Xenopus* oocytes.

**Mutant**	**EC_50_ (μM)**	***pEC*_50_**	**n_**H**_**
WT	1.7	5.76 ± 0.05	1.8 ± 0.3
Y441A	1.0	5.98 ± 0.10	1.0 ± 0.1
D238A		NF	
K255A	3.3	5.48 ± 0.06	1.9 ± 0.5
K255L	1.8	5.74 ± 0.05	1.4 ± 0.2
K255Q	1.2	5.93 ± 0.04	2.1 ± 0.4
C290A	4.0	5.40 ± 0.15	0.5 ± 0.1

*Values are mean and SEM, n ≥ 3*.

**Figure 10:**
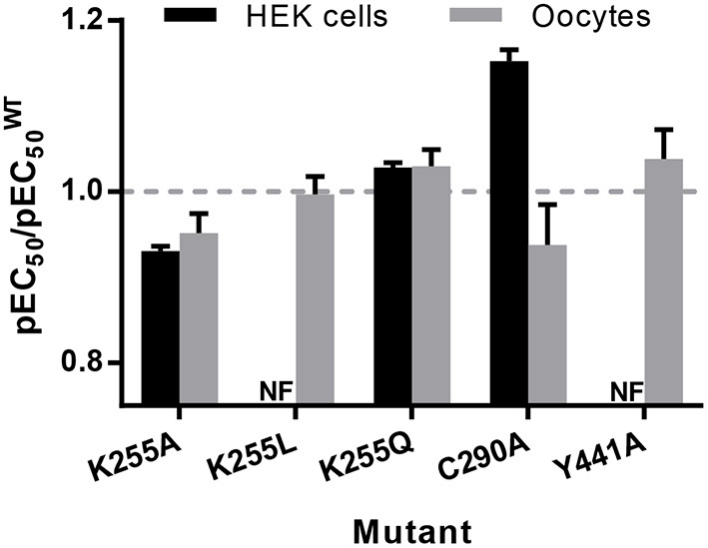
pEC50 relative to WT for mutants expressed in HEK293 cells and *Xenopus* oocytes, from Tables 1–3. Data is mean ± SEM, *n* ≥ 3; values less than 1 indicate loss-of-function, and values greater than 1 indicate gain-of-function.

On expression in *Xenopus* oocytes, both Y441A and K255L (which were non-responsive in HEK cells (Mesoy et al., 2019, Table 2)) gave WT-like responses (Figure 9 and Table 3). This demonstrates that Y441, which is required for channel function in HEK293 cells, is not in *Xenopus* oocytes. For the mutants that did function in HEK cells, we observed two different patterns in oocytes. While K255A showed similar shifts relative to WT in both expression systems, C290A was more sensitive to ligand than WT in HEK cells but less sensitive in *Xenopus* oocytes (Figure 10).

To assess the impact of lipid composition on channel function, we investigated the effects of lipids on Y441 and nearby residues in the simulated WTR and MR. The two models make use of the same lipid composition (POPC, POPE and cholesterol), but the local lipid environment around each of the five subunits differs due to varied lipid diffusion during the simulation. We found no difference between the two models in any of the measures described below (distributions of lipids around Y441, relative positions of lipids within the membrane with respect to Y441, and hydrogen bonds formed with residues 441 and/or 238).

**Figure 11:**
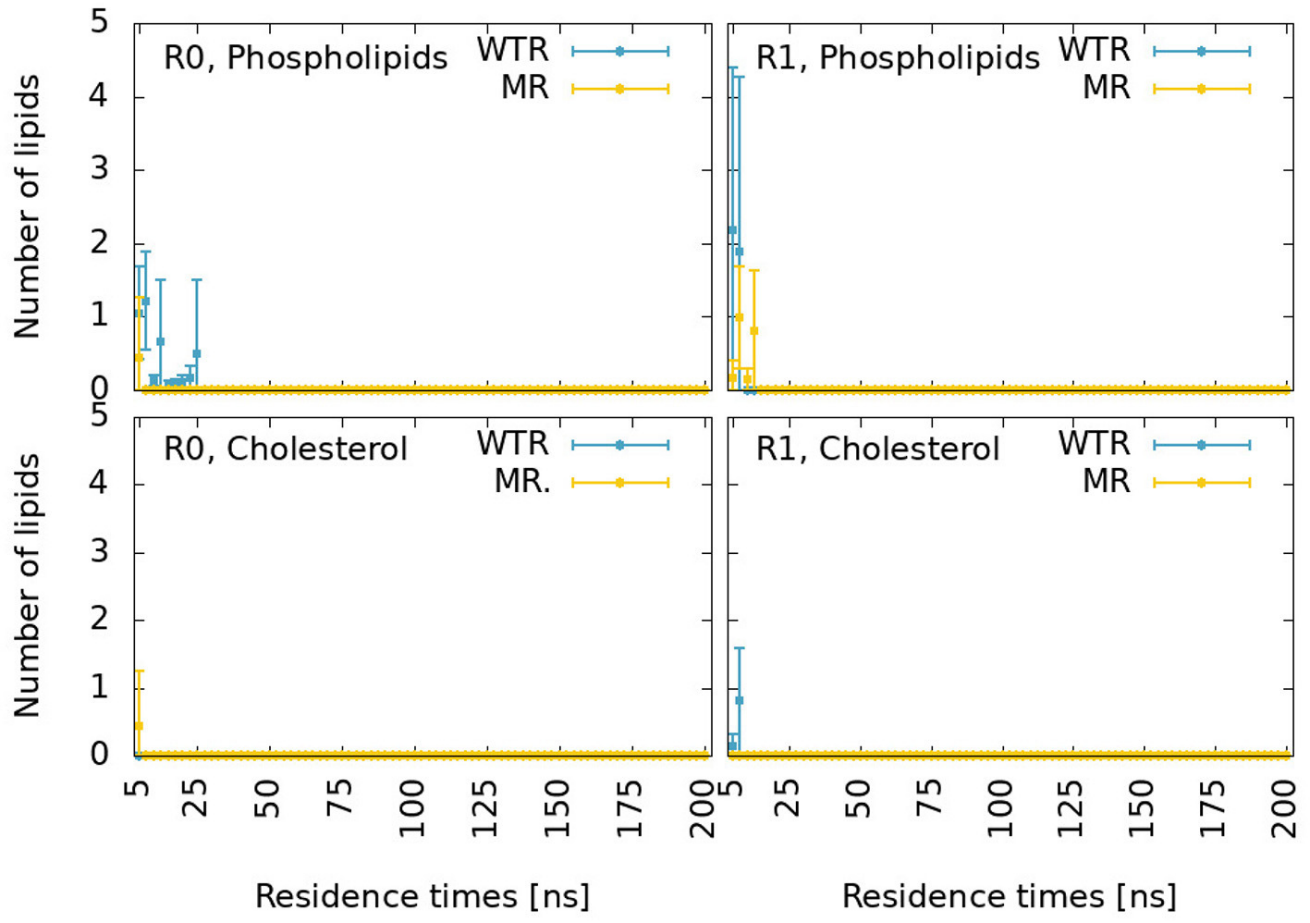
Proximity lifetime distributions of any phospholipid **(top)** and of any cholesterol **(bottom)** in close proximity to residue 441 in the WTR (blue) and in the MR (yellow) for replicas R0 **(left)** and R1 **(right)**. The results are shown for residence times larger than 5 ns.

In order to evaluate the fitness of residue 441 to give rise to a valid lipid binding site, we first evaluated the proximity lifetime distributions of lipids around this residue, for the two replicas (R0 and R1) separately as this quantity strictly depends over the specific replica. The results are reported for phospholipids and cholesterol in Figure 11. POPC and POPE are grouped together, since 441 is at the level of phospholipid tails, which are indistinguishable for POPC and POPE. For this calculation, we considered multiple time windows, shifted by 5 ns, and evaluated averages and standard deviations for each 5 ns time period. The statistics available for each residence time decreases for larger times.

These distributions are characterized by very fast decays. While cholesterol only exhibits one binding event for around 15 ns in R1 for the WTR, phospholipids display some binding events up to 25 ns. However, no event is seen for any binding duration beyond this value, meaning that possibly all interactions at the level of residue 441 are relatively weak.

We calculated the *z* component (where *z* is the parallel direction to the protein axis) of the distance of center of mass of lipids selections from the center of mass of the five 441 residues, shown in Supplementary Figure 5. Residue 441 is at the same height as the center of mass of cholesterol molecules and phospholipid tails of the lower (inner) leaflet. Phospholipid tails may disrupt interactions between residue 441 and D238, and could intercalate within the subunit (Crnjar and Molteni, 2020). Phospholipid heads are on average quite far from residue 441, but might still form sporadic hydrogen bonds with this residue or with D238. Cholesterol could form π-π interactions with the side chain of residue 441 when present (i.e., in the WTR), sporadic hydrogen bonds with either residue 441 or D238, or conversely could intercalate within the subunit (possibly aided by the lack of the side chain of residue 441 in the MR). A previous *in-silico* study observed cholesterol interacting with Y441 by means of π-π interactions as well as hydrogen bonds (Guros et al., 2020).

**Figure 12:**
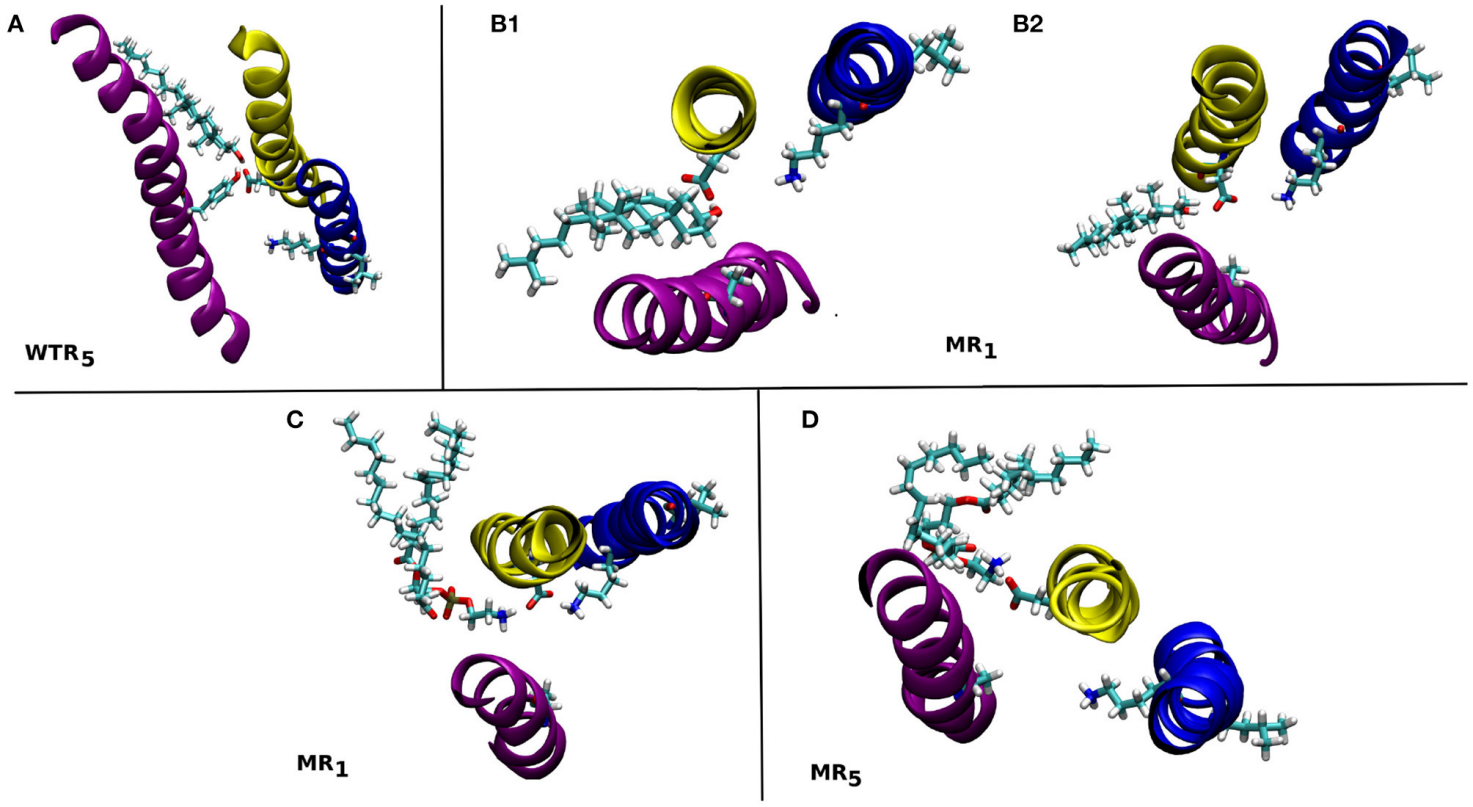
Snapshots from the MD simulations showing some of the lipids that form hydrogen bonds with residues 441 and/or 238 in the WTR **(A)** and MR **(B–D)**. These are discussed in the text. M1: yellow, M2: blue, M4: purple.

The hydrogen bonds formed between residue 441 (or D238) and lipids, calculated over R01-400 for the two models, are displayed in Supplementary Table 2 and depicted in Figure 12.

Only isolated and weak hydrogen bonds were observed between lipids and residues 441 and 238. While their average values are low (or even zero), their effects are still interesting. In the WTR, subunit 5 (Figure 12A), a POPE molecule formed hydrogen bonds with both Y441 and D238, pulling D238 away from K255. In the MR, a cholesterol molecule was observed making hydrogen bonds in subunit 1 that pushed D238 outward and away from K255 (Figure 12B_1_), but this did not result in a pulling of D238 at other timepoints (Figure 12B_2_). Similarly, a POPE molecule in subunit 1 engaged in hydrogen bonds with D238 that did not prevent it from also interacting with K255 (Figure 12C), but another POPE, in subunit 5, instead pulled D238 outward when interacting with it (Figure 12D). Overall, no lipid interactions in this region were observed to promote or inhibit intrasubunit interactions at the level of Y441, nor to prefer any particular conformation or interaction over any other, although further work beyond the scope of this study is required to confirm this, e.g., by using coarse-grained methods, in order to produce a larger statistics of possible lipid interactions with or near Y441.

## 4. Discussion and Conclusions

The aim of this work was to probe the effects of the M4 helix in 5-HT_3A_R function using the non-functional Y441A mutation. Our data suggest that Y441 connects to K255 on the pore-lining M2 helix via the M1 residue D238, and that this interaction is necessary for receptor function in HEK cells but not in *Xenopus* oocytes. We found no indications that Y441 or Y441A-mediated uncoupling affect the M4 tip or the Cys-loop.

### 4.1. Vertical Mechanism of Connection

In our simulations of both the WTR and the MR, the C-terminal domain appeared to move independently of residue 441 (Figure 2), indicating that Y441A does not act through large-scale shifts in M4 movement. We further confirmed the independence of the CTD from the Y441 by dynamical correlation measurements (Figure 3), and found no differences in hydrogen bonds from residues other than 441 between the two models (Figures 4, 7). From this we conclude that despite the structural appeal and the early indications pointing to a vertical mechanism of action, M4-mediated coupling does not occur via interactions of the M4 tip with the Cys-loop, as the CTD of M4 is unaffected by the mutation that disconnects channel opening from ligand binding.

### 4.2. Radial Mechanism of Connection

The possibility of a radial mechanism of connection from M4 to the channel pore is appealing: substitution of a sufficiently different residue for Y441, D238, or K255 individually abolished receptor function but not ligand binding in HEK293 cells (Mesoy et al., 2019, Tables 1, 2). However, these substitutions are not equal. The aromatic group of Y441 must be key to its function, as Y441F has WT-like function where Y441A does not. Likewise some specific property of D238 is required for correct channel function, as evidenced by the lack of function of D238A. K255 is subtly different: the data suggest the residue at this position must be polar or charged if it is large: K255E, C, and Q are all functional, but K255L—bulky and uncharged—results in non-functional receptors. However, a large polar residue is not strictly required here, as evidenced by the WT-like function of K255A.

We propose that a major requirement of residue 255 may be to allow the displacement of M2 on channel opening. The movement of M2 on channel opening in 5-HT3A has been described as a rotation and outward displacement (Basak et al., 2018b). Polovinkin et al. show an outward movement of M2 (Polovinkin et al., 2018), especially the lower half, as well as a rotation to clear the restricting L9' (L260) residues from the center of the pore. We propose that residue 255 requires a polar character here to allow this outward movement if the residue is large (hence K255L blocking channel opening), perhaps involving the observed hydrogen bond to D238 (Figure 7). However mutation of K255 to a small residue like alanine may also allow outward movement of this helix, explaining the mutation pattern here.

Conversely the removal by alanine mutation of either Y441 or D238 abolishes channel function. This, too, may be related to the movement of M2 on channel opening (and hence explain why these mutations prevent it). Figure 2C in Basak et al. (2018b) and the “morph” videos in Polovinkin et al. (2018) show particularly well the movement of M1 and M4 on channel opening—outward and, for M4, upward. It seems likely that Y441 and D238 may be required for this movement of their respective helices, and that this helical movement is required for the outward channel-opening movement of M2 discussed above.

While each residue may act individually, it is also striking that these three residues in close proximity give the same functional phenotype on mutation. A Y441-D238 interaction in particular is likely to be required for channel function, though a hydrogen bond is not (as Y441F is WT-like). K255 is not specifically required, though a large non-polar residue at this location is disruptive. While K255A does function, the requirement for any larger residue at position 255 to have some polarity—be it a positive charge, a negative charge, or only a polar group—does point intriguingly toward the hydrogen bond noted with D238 (Figure 7). Determining precisely which of these residues interact and how will be key to understanding the wider mechanism of channel opening in 5-HT_3_R receptors.

### 4.3. Rescue of Non-functional Receptors

The stark difference between the uncoupled state of Y441A and K255L in HEK293 cells and their WT-like behavior in *Xenopus* oocytes is intriguing. Putting this in the context of the literature, a wider pattern emerges where mutations in cationic pLGICs M4 helices that affect EC50 are slightly beneficial in *Xenopus* oocytes, but detrimental in HEK293 cells:

Many (11 out of 24) alanine mutations in the α7 nAChR M4 helix improve function in oocytes (da Costa Couto et al., 2020). In contrast, several (8 out of 27) alanine mutations in the α4β2 nAChR M4 helix abolish function (but not ligand binding) when expressed in HEK293 cells (Mesoy and Lummis, 2021). Looking only at the C-terminal end of the M4 helix, it seems that while it can be deleted without ablating function in both ELIC (Hénault et al., 2015) and the *Torpedo* nAChR (Tobimatsu et al., 1987), deletion or alanine mutation of individual C-terminal residues abolish function in the 5-HT_3A_R and in the α4β2 nAChR (Pons et al., 2004; Butler et al., 2009; Mesoy et al., 2019; Mesoy and Lummis, 2021). However we note that the ELIC and nAChR studies were performed in *Xenopus* oocytes, and the other four in HEK293 cells, indicating that the requirement for the C-terminal domain may be more a function of the expression system than of the specific channel. An exception to this pattern is that alanine mutations in the M4 of the α subunit of the muscle nAChR expressed in oocytes show both gains and losses of function (Thompson et al., 2020). We note that these mutations are only present in 2 out of 5 subunits per channel, and what would happen in a muscle AChR with all 5 positions mutated is as yet unknown.

This variation in channel function of M4 mutants between expression systems has not been observed in anionic or bacterial channels. In anionic channels, alanine mutations (especially of aromatic residues) are generally detrimental to channel function, regardless of expression system (Haeger et al., 2010; Cory-Wright et al., 2018; Tang et al., 2018). Mutations in M4 have opposite effects in two bacterial pLGICs assayed in the same system (*Xenopus* oocytes): Many (15 out of 25) alanine mutations in the GLIC M4 are detrimental to channel function, while a majority (26 out of 31) of alanine mutations in the ELIC M4 improve channel function (Hénault et al., 2015).

We suggest that there exists a functional mechanism in cationic pLGICs requiring the M4 helix (including the C-terminus) which is necessary in HEK cells but not in *Xenopus* oocytes. If so, firstly conclusions about M4 function from studies in *Xenopus* oocytes cannot be extended to other expression systems, specifically not HEK cells, and vice versa. Secondly, this would indicate that some factor is either present in oocytes that can rescue these mutants, or present in HEKs that inhibits them. Due to the locations of these mutations in the lipid bilayer, we suggest that this is unlikely to be an intracellular factor or a post-translational modification. The proximity of these mutations to the lipid bilayer, along with the wide range of previously characterized lipid-uncoupled receptors, points us to the hypothesis that some element of the lipid bilayer of *Xenopus* oocytes is able to compensate for the absence of Y441. A speculative but attractive option, given the sensitivity of pLGIC activity to the local lipid environment, is that the *Xenopus* “rescue” factor could be cholesterol. Adding cholesterol (and/or negatively charged phospholipids) to reconstituted membranes promotes pLGIC function (Fong and McNamee, 1986; Baenziger et al., 2000). The importance of cholesterol in particular is highlighted by the fact that increasing the percentage of cholesterol in a reconstituted membrane increases the number of nAChRs that open on agonist binding (Rankin et al., 1997).

With regards to our simulations, an inhibitory factor in HEK cells would not necessarily be visible; indeed observed interactions and binding events depend on the local lipid environment around each of the five subunits and can only provide a hint of the role of the involved lipid species. Care must also be taken when comparing experiments and simulations, since the former were carried out at room temperature and the latter at body temperature (in order to keep the modeled membrane above the gel transition temperature of all lipids present).

We cannot as yet, however, rule out an endogenous or exogenous factor which does not act on Y441 itself. From our simulations, we were able to conclude that only sporadic lipid binding events or interactions with D238 occurred at the level of Y441 (Figure 11), and that no promotion/inhibition of the radial mechanism was unambiguously observed by lipid molecules. Given that the cholesterol content has been shown to increase the chances of any lipid binding event in the 5-HT_3A_R receptor (Crnjar and Molteni, 2020), we can speculate that the oocyte membrane, with higher cholesterol content, could promote additional binding events near residue 441.

In conclusion, we have thoroughly investigated the role of a point mutation (Y441A) in the 5-HT_3A_R M4 helix, using it as a proxy for the role and function of the entire helix in coupling ligand binding to channel opening, using both *in-silico* techniques and experiments in two different expression systems. We showed that Y441-mediated coupling involves D238 on M1 and K255 on M2, creating a radial chain from the channel pore to the lipid-facing M4. No effect is propagated vertically from Y441 toward the M4 tip or the Cys-loop, leading us to conclude that Y441-mediated coupling specifically, and M4-mediated coupling in general does not depend on M4/Cys-loop interactions.

Finally, we speculate that the rescue of uncoupled mutants in *Xenopus* oocytes may be due to the lipid composition of the oocytes, and suggest cholesterol as a potential candidate for rescuing receptors that are non-functional yet expressed in HEK cells.

## Data Availability Statement

The raw data supporting the conclusions of this article is available at: http://doi.org/doi:10.18742/RDM01-701.

## Author Contributions

AC, SM, SL, and CM participated in the research design and wrote and contributed to the manuscript. AC performed the simulations. SM conducted the experiments. AC, SM, and SL performed data analysis. All authors contributed to the article and approved the submitted version.

## Conflict of Interest

The authors declare that the research was conducted in the absence of any commercial or financial relationships that could be construed as a potential conflict of interest.
