# Supplementary material for: A Single Mutation in the Outer Lipid-Facing Helix of a Pentameric Ligand-Gated Ion Channel Affects Channel Function Through a Radially-Propagating Mechanism
Source: Front Mol Biosci. 2021 Apr 30;8:644720. doi: 10.3389/fmolb.2021.644720 (PMC8119899; doi:10.3389/fmolb.2021.644720)
Supplement: Supplementary file 1 [file Data_Sheet_1.PDF]

Supplementary Material: A Single Mutation in The Outer Lipid-Facing Helix of a Pentameric Ligand-Gated Ion Channel Affects Channel Function through a Radially-Propagating Mechanism

1 SEROTONIN PARAMETRIZATION

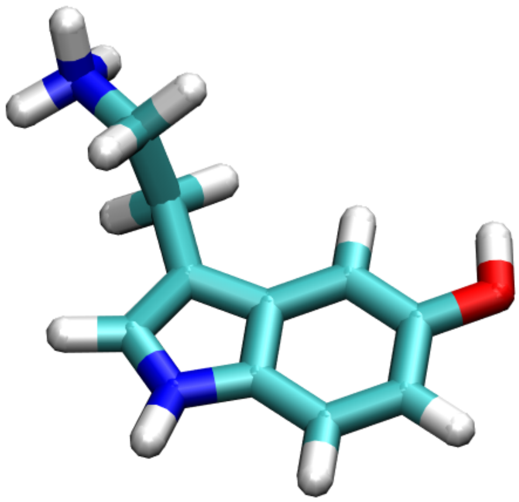

Figure S1. Representation of a protonated serotonin.

As a non-standard residue, serotonin (5-HT), represented in Fig. S1, was parametrized according to the general AMBER force field (Wang et al. (2004)), while its partial charges were evaluated with *ab-initio* calculations, performed with the software Gaussian09E (Frisch et al. (2013)), The serotonin binds to the receptor in its protonated form, i.e with a terminal, positively charged amine group NH_3 (shown in Fig. S1). In order to account for the rotations of the serotonin hydroxyl group and for the flexibility of its tail, the partial charges were calculated for twelve configurations, each considering a different orientation of the aforementioned groups. Each of these conformations was optimized at the density functional theory level with the b3lyp exchange and correlation functional (Becke (1993)) and a 6-31G* basis set. A single point energy calculation was then carried out on the optimized geometry at the Hartree Fock level with the same 6-31G* basis set (for consistency with the AMBER force field parametrization). A multiconfigurational RESP fitting was then performed for the calculation of the partial charges, by making use of the 12 configurations (Bayly et al. (1993)).

2 PROTEIN EQUILIBRATION PROTOCOL

Restraints	Duration
25 $kcal/(mol\mathring{A}^2)$ on protein α carbons and 5-HT rings	50 ns
Force constant rescaling from 25 to 15 $kcal/(mol\mathring{A}^2)$	3 ns
15 $kcal/(mol\mathring{A}^2)$ on protein α carbons and 5-HT rings	50 ns
Force constant rescaling from 15 to 8 $kcal/(mol\mathring{A}^2)$	4 ns
8 $kcal/(mol\mathring{A}^2)$ on protein α carbons and 5-HT rings	10 ns
Force constant rescaling from 8 to 4 $kcal/(mol\mathring{A}^2)$	4 ns
4 $kcal/(mol\mathring{A}^2)$ on protein α carbons and 5-HT rings	10 ns
Force constant rescaling from 4 to 1 $kcal/(mol\mathring{A}^2)$	7 ns
1 $kcal/(mol\mathring{A}^2)$ on protein α carbons and 5-HT rings	10 ns
	Total: 148 ns

Table S1. Full equilibration procedure of both wild-type receptor and mutated receptor models.

3 M4 SEQUENCE

M4 helix sequence as found in the PDB entry 6DG8 (from the N-terminal to the C-terminal): WLRVGYVLDRLLFRIYLLAVLAYSITLVTLWSIWHYS.

4 PROTEIN AND M4 STABILITY

The stability of the protein, and of the M4 helices in particular, is demonstrated by the root mean square displacements (RMSDs) of the backbone atoms with respect to the last step of the equilibration protocol outlined in Table S1, as a function of time, for both the wild-type receptor (WTR) and the mutated receptor (MR) and in both replicas R0 and R1. The graphs prove that the simulated models were stable over the investigated time-windows.

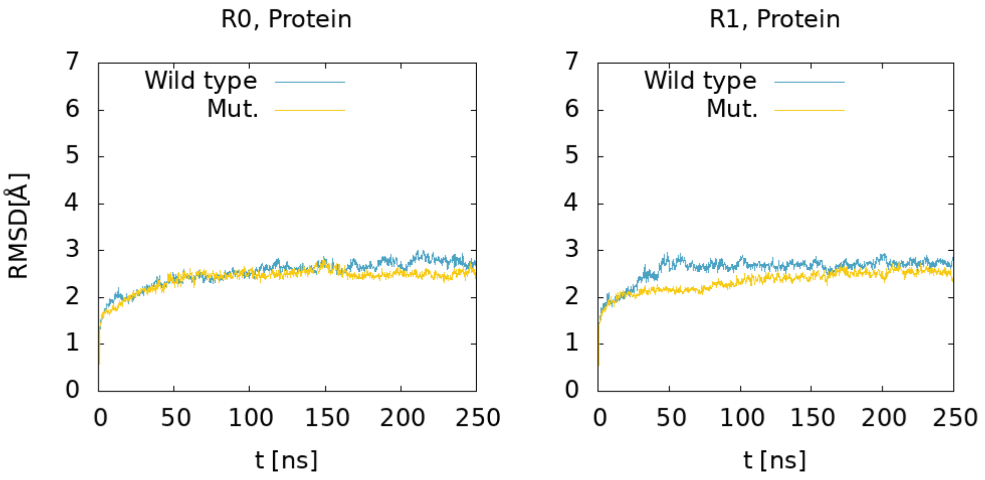

Figure S2. RMSD of protein in models WTR (blue) and MR (yellow) and in replicas R0 and R1.

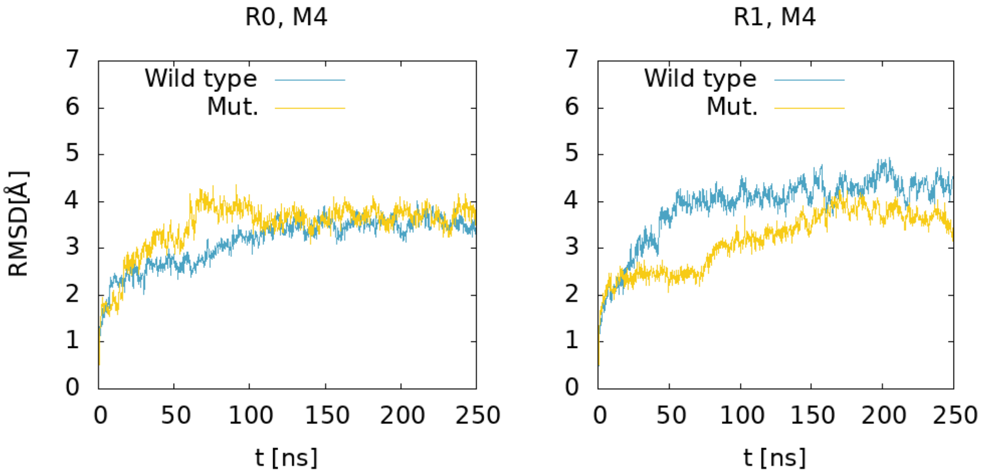

Figure S3. RMSD of M4 helices in models WTR (blue) and MR (yellow) and in replicas R0 and R1.

5 MEMBRANE HEIGHT WITH RESPECT TO TRANSMEMBRANE DOMAIN

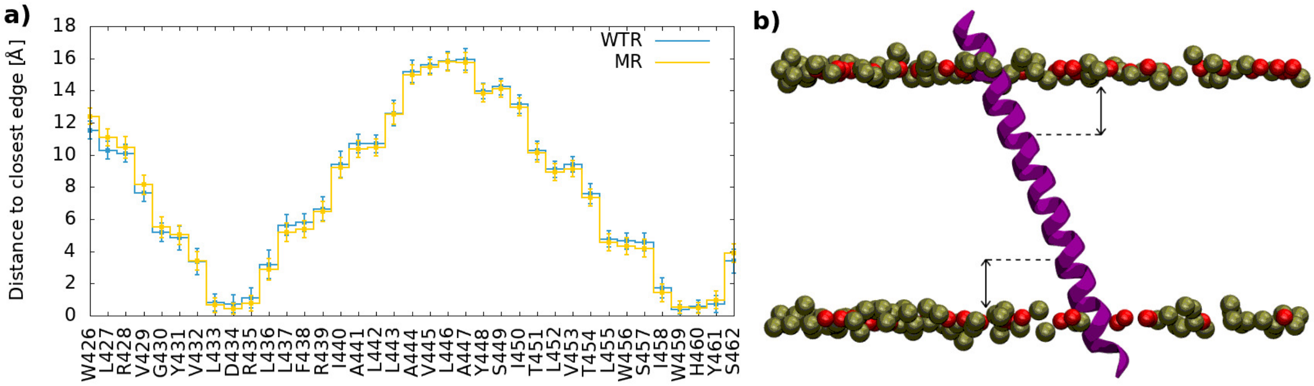

Figure S4. a) average z component of the distance between the center of mass of the ring given by a certain M4 residue in the five subunits and the nearest layer of lipid heads (phosphorus for POPC and POPE, oxygen for cholesterol). This is calculated, for all M4 residues, over R01-400, for the WTR (blue) and the MR (yellow). b) Schematic representation of the calculated quantity.

6 LIPIDS DISTANCE ALONG Z FROM RESIDUES 441 AND 448

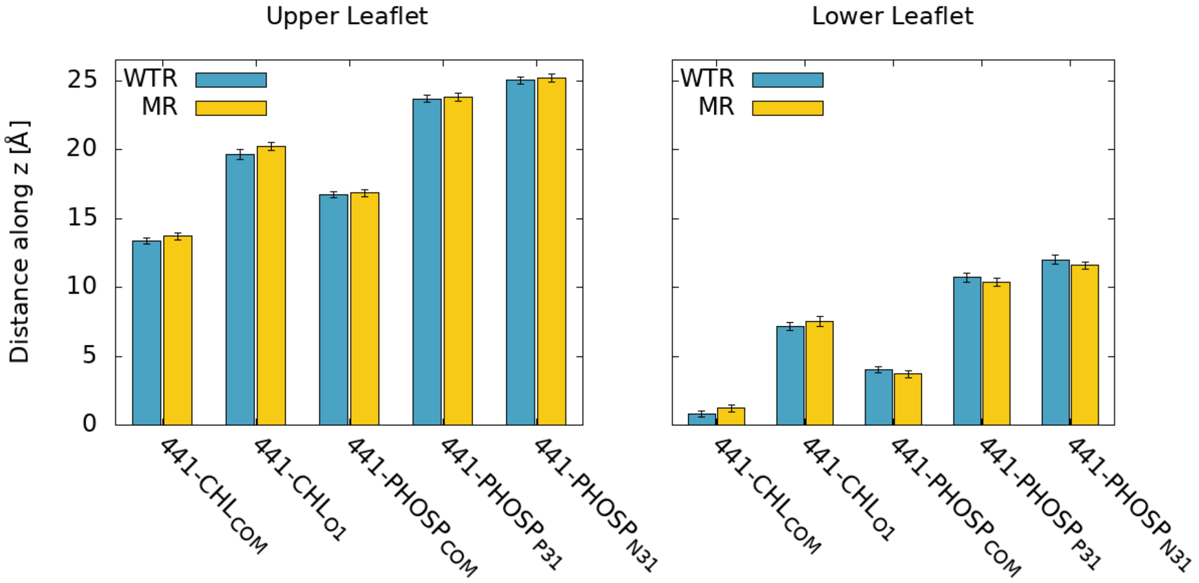

Figure S5. z component of the distance of the center of mass of the five 441 residues from the center of mass of lipids selections (of the upper and lower leaflet): cholesterol center of mass (COM), cholesterol oxygen (O1), phospholipid (PHOSP) center of mass (COM), phospholipid phosphorus (P31), and phospholipid head nitrogen (N31). The results are shown for R01-400 for the WTR (blue) and the MR (yellow).

7 HYDROGEN BONDS BETWEEN LIPIDS AND RESIDUES 441 AND 238

Model	Hydrogen bond	Value
**WTR**	D238-CHL_5 a)	0.2 ± 0.4
	Y441-CHL_5 a)	0.0 ± 0.2
**MR**	D238-CHL_1 b_1), b_2)	0.0 ± 0.2
	D238-$POPE_1$ c)	0.1 ± 0.3
	D238-$POPE_5$ d)	0.0 ± 0.2

Table S2. Hydrogen bonds formed between residue 441 (or D238) and lipids. Errors are calculated as standard deviations over time-data, while subscripts refer to subunits.

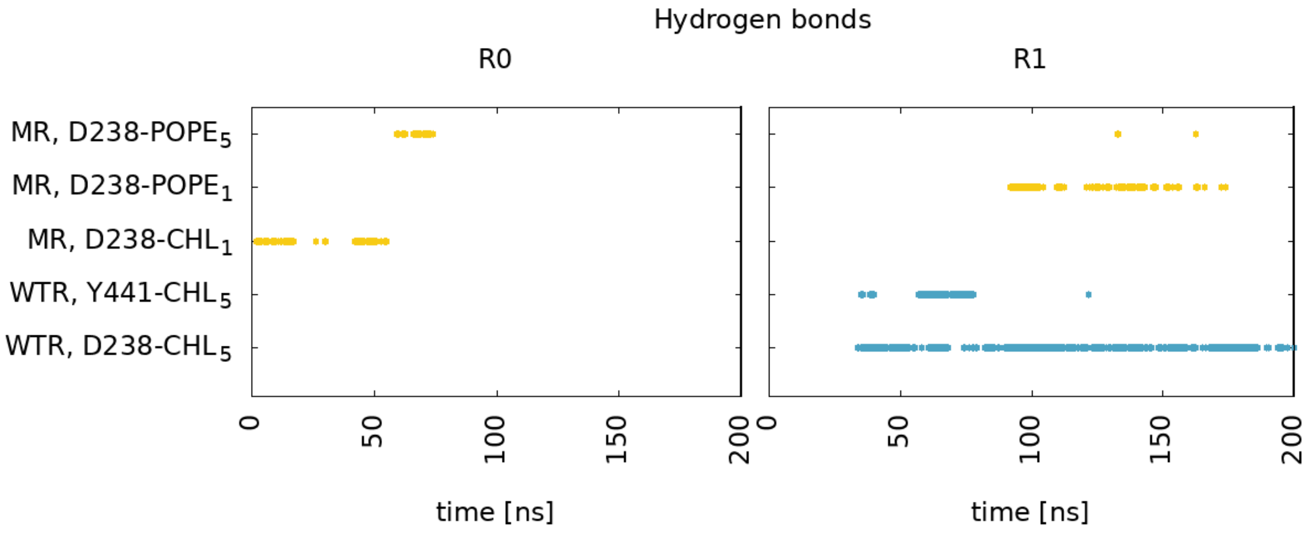

Figure S6. Hydrogen bonds between residue 441 (or D238) and lipids, in replicas R0 and R1.

8 K255-D238 SIDE CHAIN DISTANCES OVER TIME

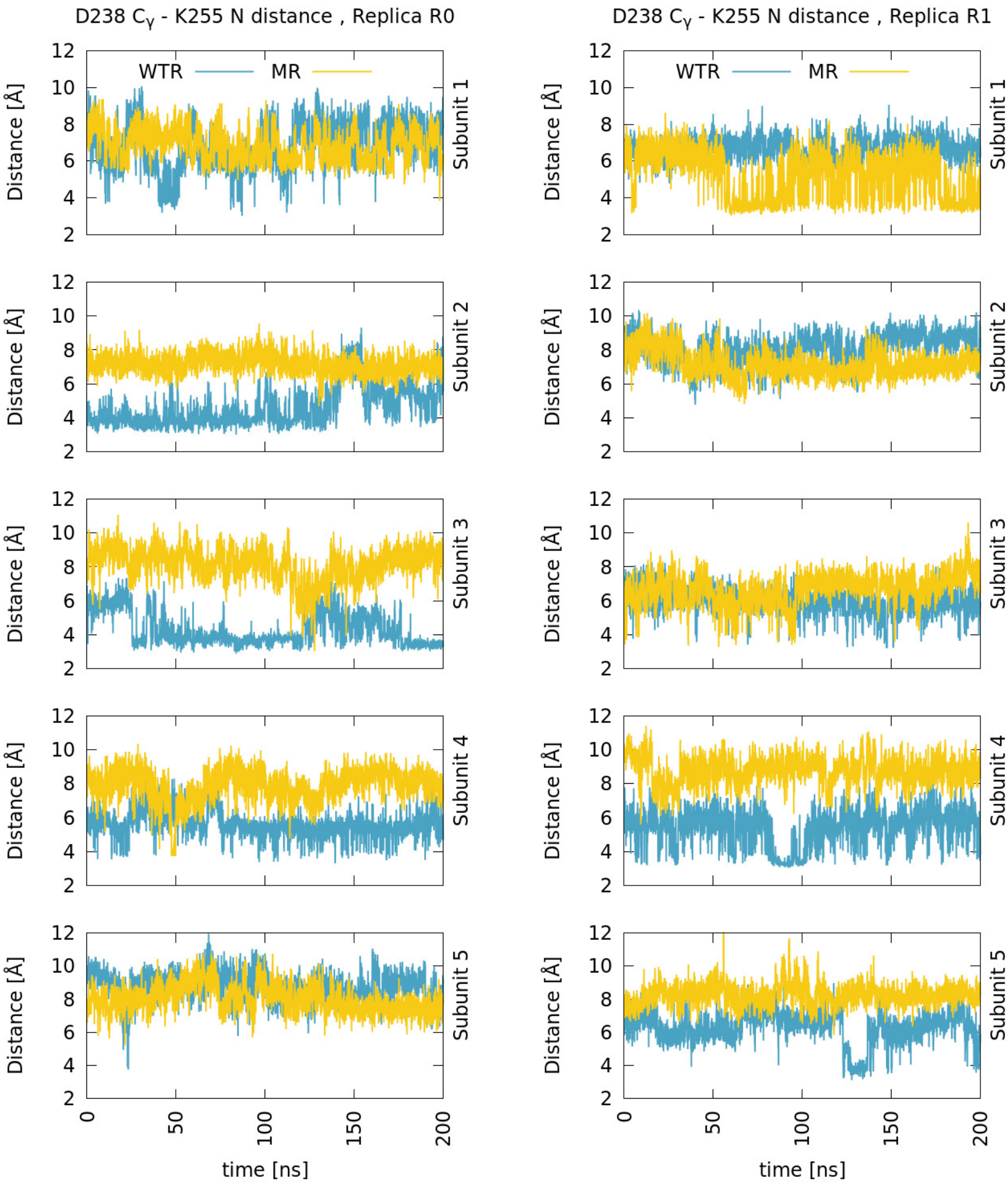

Figure S7. Time evolution of D238 C_γ to K255 side chain nitrogen distances, in replicas R0 and R1, for the WTR (blue) and the MR (yellow) in the five subunits.

REFERENCES

Bayly, C. I., Cieplak, P., Cornell, W., and Kollman, P. A. (1993). A well-behaved electrostatic potential based method using charge restraints for deriving atomic charges: the resp model. *The Journal of Physical Chemistry* 97, 10269–10280. doi:10.1021/j100142a004

Becke, A. D. (1993). A new mixing of hartree–fock and local density-functional theories. *The Journal of Chemical Physics* 98, 1372–1377. doi:10.1063/1.464304

[Dataset] Frisch, M. J., Trucks, G. W., Schlegel, H. B., Scuseria, G. E., Robb, M. A., Cheeseman, J. R., et al. (2013). Gaussian 09, revision e.01

Wang, J., Wolf, R. M., Caldwell, J. W., Kollman, P. A., and Case, D. A. (2004). Development and testing of a general amber force field. *Journal of Computational Chemistry* 25, 1157–1174. doi:10.1002/jcc.20035
